# Volatile Organic Compound Emissions From Heated Synthetic Hair: A Pilot Study

**DOI:** 10.1177/1178630219890876

**Published:** 2020-01-29

**Authors:** Donna Auguste, Shelly L Miller

**Affiliations:** 1 ATLAS Institute, University of Colorado Boulder, Boulder, CO, USA; 2 Department of Mechanical Engineering, Environmental Engineering Program, University of Colorado Boulder, Boulder, CO, USA

**Keywords:** VOC, braiding, African American, asthma, personal care products, indoor air quality

## Abstract

Volatile organic compounds (VOCs) are emitted from a variety of household and personal care products. Many VOCs are known to be potentially toxic or carcinogenic. Synthetic hair is used in hair-styling practices, including practices in African American communities that involve singeing or heating the synthetic hair. The research questions that we sought to answer were as follows: Are VOCs emitted from singed or heated synthetic hair? If so, what are the VOC species and relative masses identified in singed or heated synthetic hair? We tested samples from 2 sources of singed and heated synthetic hair in a microchamber; one source was flame-retardant synthetic hair and the other source was non-flame-retardant synthetic hair. Our findings confirmed that VOCs are emitted from singed or heated synthetic hair for both types of sources. For flame-retardant synthetic hair, we identified and measured mass for species that included acetone, acetonitrile, 2-butanone, benzene, chloromethane, chloroethane, and 1,2-dichloroethane. For non-flame-retardant synthetic hair, we identified and measured mass for species that included acetone, acetonitrile, chloromethane, trichlorofluoromethane, and 2-propanol.

## Introduction

**Figure 1.**
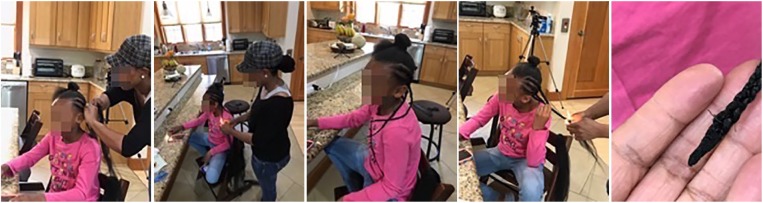
Photographs of volunteers demonstrating a typical braid-burning practice that is used during hair braiding to seal the tips of synthetic braids and prevent unraveling. Used with permission (Auguste et al, in preparation, 2020).

During a research project in a major municipality in the western United States, involving African American families with asthmatic children learning life-relevant data science, participants used a community/citizen science approach to collect residential indoor air quality data (Auguste et al, in preparation, 2020; Auguste, 2019).^1^ The participant families and researchers identified possible asthma triggers by measuring volatile organic compound (VOC) emissions with consumer-oriented indoor air quality sensor devices. They identified synthetic hair as a possible VOC source when used as part of a hair-styling practice in which stylists burn the braided synthetic hair with a butane lighter or heat it with an electric flatiron at temperatures up to 260°C. This braid-burning practice, shown in Figure 1, potentially releases VOCs into the air, in close proximity to the breathing zones of the stylist and the person whose hair is being braided. This is important because the health risks of VOC exposure may be significant, especially in communities with a high prevalence of asthma such as African American communities.^2,3^ Therefore, the research questions arose: Are VOCs emitted from singed or heated synthetic hair? If so, what are the VOC species and relative masses identified in singed or heated synthetic hair? We did not find answers in the literature, so we tested samples of synthetic hair under laboratory conditions to answer the questions. This article reports our initial findings.

The US Environmental Protection Agency^4^ (EPA) confirmed, in a 1989 report, that people in the United States spent approximately 90% of their time indoors. They reported on health effects of indoor pollutants, such as VOCs, emphasizing that exposure to indoor pollutants occurred in complex co-occurring mixtures, not in isolation.^5^ Exposure and risks associated with indoor air contaminants were noted as especially challenging to mitigate because sources may be enabled by occupant activities.

Indoor air pollutants may include VOC emissions from a variety of sources, such as the use of household and personal care products. We reviewed a number of research studies about indoor air quality impacts of household and personal care product use, but not synthetic hair. Dinh et al^6^ examined the emission characteristics of household product use such as hair spray, dishwashing detergent, and air fresheners, finding that in spray products, “21.6-96.4% of the VOCs were propane, iso-butane, and n-butane, which are the components of liquefied petroleum gas.” Nematollahi et al^7^ identified 228 different VOC emissions from fragranced baby products, finding the most common were limonene, acetaldehyde, ethanol, α-pinene, linalool, β-myrcene, acetone, and beta-pinene. Some researchers examined personal care products used in professional settings, such as hair salons. In those settings, VOCs and other airborne contaminants from multiple sources may mingle together to collectively affect the indoor air quality. De Gennaro et al^8^ used a thermal desorption methodology to analyze hair salon VOCs emitted from hair lacquer, shampoo, balms, hair masks, and hair dye. They found that the types of products contributing to the VOC concentrations had a more significant impact on the concentration levels than did efficiency of air exchange or number of customers. Lamplugh et al^9^ examined indoor air quality in 6 nail salons, finding concentrations of formaldehyde in 1 salon exceeding recommended exposure limits and finding health risks associated with long-term exposure. Zhong et al^10^ also examined VOCs in 17 nail salons. They measured personal inhalation exposure using passive samplers attached to clothing, and they measured area exposure using passive samplers in a backpack. They analyzed 35 nail salon products with a static headspace gas sampling method. All collected samples were analyzed with GC-MS protocols. They found that personal inhalation exposure to VOCs was 1.2 to 2.0 times higher than area exposure. They identified specific nail salon products that contributed to VOC measurements of ethyl acetate, propyl acetate, butyl acetate, n-heptane, and toluene found in most of the 17 nail salons. Hadei et al^11^ measured concentrations of benzene, toluene, ethylbenzene, xylene, formaldehyde, and acetaldehyde in beauty salons, finding possible cancer risks from benzene, formaldehyde, and acetaldehyde. However, none of these researchers looked at synthetic hair.

Using various methods and considering diverse sources, other researchers have sought to measure VOC exposure. Delfino et al^12^ measured VOCs in exhaled breath, with 21 asthmatic children who lived near high-traffic areas. They identified benzene methylene chloride, styrene, toluene, o-xylene and m,p-xylene, tetrachloroethylene, and p-dichlorobenzene in more than 75% of the exhaled breath samples collected and associated those with combustion-related sources. Boyle et al^13^ used urine analysis to measure exposure to 28 VOC metabolites for pregnant women and identified exposure sources in their homes such as “air fresheners, aerosols, paint or varnish, organic solvents, and passive/active smoking.” Hoang et al^14^ measured VOC levels in 34 early childhood education environments. Their data collection methods included use of active sampling into sorbent tubes and the EPA TO-17 thermal desorption GC-MS analysis. They identified levels of benzene, chloroform, ethylbenzene, and/or naphthalene exceeding the limits established by the state of California for carcinogenic risk. They noted the need to identify sources of the VOCs, and the need for developing non-occupational health benchmarks for children. The quantitative structure-activity relationship models they developed may be useful for establishing such benchmarks in future work.

Although synthetic hair is used for a variety of hair styles, heating synthetic hair as part of a braided hair-styling practice is notably a cultural expression in communities associated with African ethnicities. We searched the literature for research about health impacts of hair care choices. We read, for example, the work by Tanus et al,^15^ which emphasized the importance of doctors being familiar with the hair care practices of women of African ethnicities, to understand and advise them about health impacts of hair care choices. This research encouraged a respect for hair care practices as a form of personal and cultural expression. It included description and discussion of potential health impacts of hair-braiding practices with synthetic hair, such as traction alopecia, but did not specifically discuss singeing or heating synthetic hair, or emissions from that styling practice.

Recall that our original concern about whether or not heated synthetic hair emits VOCs is because of the possible adverse health impact for African American family members with asthma. The Centers for Disease Control^2^ has documented that asthma affects African American and Puerto Rican people in the United States in disproportionately high numbers. There are 26 million people in the United States with asthma. Approximately 10.3% and 13.7% of African Americans and Puerto Ricans have asthma, respectively; prevalence rates in all other demographic groups are less than 8%. Exposure to potential asthma triggers in these communities may have significant and serious health impacts. Chin et al^16^ specifically studied VOC levels in the homes of asthmatic children. They found that the highest VOC concentrations were d-limonene, toluene, p,m-xylene, and ethyl acetate. They identified emission sources that included cigarette smoking, solvent-related emissions, household products, and pesticides, but did not include synthetic hair sources. Martin et al^17^ noted that VOCs were among the home asthma triggers that may “constitute physical stressors that can lead to airway hyper-reactivity and allergen-induced airway inflammation resulting in asthma morbidity and increased burden of disease,” which suggests that it is important to know whether synthetic hair is emitting VOCs, especially near the breathing zones of asthmatic participants in the hair-styling practice.

To determine whether VOCs are emitted from heated synthetic hair, we used an environmental chamber to extract airborne emissions from synthetic hair samples. Environmental scientists and engineers are increasingly making use of microchambers as analysis instruments, and specifically for working with heated samples. Kamarulzaman et al^18^ successfully used microchamber analysis to identify VOCs from rubber materials. Schieweck and Bock^19^ also used microchamber analysis to extract and identify VOCs from paint. Yu and Crump^20^ tested flooring adhesives for VOC emissions.

The specific question of VOC emissions from synthetic hair as used in braided hair styles appears to be understudied. This study aims to test synthetic hair to determine whether VOCs are emitted when the synthetic hair is heated and to identify relevant species and masses if VOCs are found in the emissions. If VOCs are found in the emissions of the heated synthetic hair, confirming the informal finding of the community/citizen-science, this initial study will help researchers to identify future work for further study.

## Materials and Methods

### Study design

We designed and conducted this study to determine whether any VOCs are emitted from singed or heated synthetic hair and, if so, to test emissions to identify VOC species and mass. In Spring 2018, working in a university lab in the western United States, we collected emissions from 2 sources of singed and heated synthetic hair into sorbent tubes through a microchamber. We provided those sorbent tubes to an independent lab in the western United States, where analytes were extracted and analyzed through a nationally standardized thermal desorption–gas chromatography–mass spectrometry (TD-GC-MS) method.

### Analytic instruments

**Figure 2.**
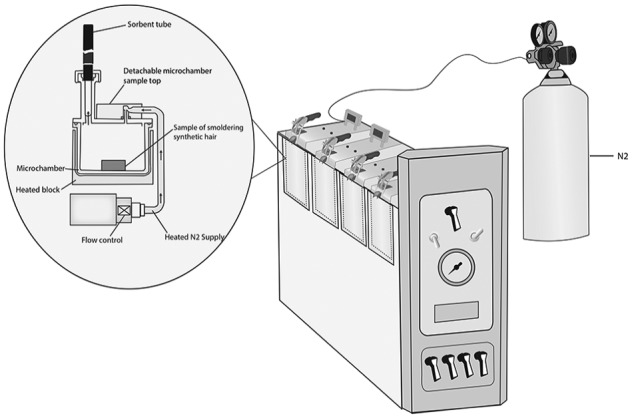
Illustration of a Markes Microchamber/Thermal Extractor™ model 250 (µ-CTE250™) with a pressure-regulated supply of high-purity nitrogen gas (N_2_), and details of an individual cylindrical microchamber. The cup that holds the sample in the cylindrical microchamber is 36 mm deep × 64 mm diameter, volume of 0.0445 mm^3^.

To prepare for our testing methodology, we set up and connected a Markes Microchamber/Thermal Extractor™ model 250 (known as µ-CTE250 and M-CTE250, with alternate spelling micro-chamber and micro chamber). The µ-CTE250, shown in Figure 2, had a bench footprint of 52 cm × 16 cm. We followed setup guidelines specified in the operator manual.^21^ We set up the µ-CTE250 in a clean wet lab environment at our University of Colorado Boulder. The µ-CTE250 that we used had four cylindrical chambers (known individually as microchambers). Each of the four microchambers was designed to hold one inert-coated stainless-steel cup, 36 mm deep and 64 mm in diameter (known as a removable microchamber pot). Each of the four microchambers had an inert-coated stainless-steel lid that we could close and clamp once a sample was placed inside of the microchamber, with or without a porcelain crucible inside the stainless-steel cup. The lid was equipped with a sampling port to which we attached Carbotrap 300^®^ sorbent tubes for emission collection. Carbotrap 300 sorbent tubes were 0.11581 L, 0.25 in. outer dimension, 3.5 in. length, stainless-steel thermal desorption tubes with a preconditioned multi-bed matrix of graphitized carbon black adsorbents (specifically, Carbotrap C, Carbotrap B, and Carbosieve SIII). We did not use an external pump.

We connected the µ-CTE250 to a high-purity nitrogen gas supply (N_2_) through a stainless-steel diaphragm cylinder head regulator and refrigeration-grade copper supply line with swage fittings, which maintains constant flow of gas through each microchamber. A set of 4 toggle switches, on the front panel, controlled the on/off flow of nitrogen gas to each individual microchamber. A single toggle switch, also on the front panel, controlled the low/high flow range. The N_2_ gas was operated at approximately 46 lb/in^2^ for each run, monitored by the pressure gauge on the front panel. The microchamber architecture, shown in Figure 2, included a heated block under the microchamber that warmed samples up to 250°C (482°F), controlled by the heater on/off switch on the front panel and cooled by the fan off/off switch on the front panel. We installed a black ultra-high-purity O-ring on each of the 4 microchambers, to accommodate temperatures up to 250°C. We used the temperature controller to set target temperatures and monitor actual temperatures in the microchambers. For the test runs that included singeing the synthetic hair, we used a hand-held butane lighter to ignite the samples.

### Cleaning

We followed cleaning guidelines specified in the operator manual, where the manufacturer noted that the inert stainless-steel coating on the microchamber pots, inserts, and lids minimizes contamination.^16^ Before each run of samples, we cleaned the removable microchamber pots and the porcelain crucibles. We washed them with Dawn^®^ dishwashing liquid, rinsed twice with distilled water, then rinsed once with isopropanol. We wiped the inside lid of each microchamber with a paper towel wet with distilled water. As a final step, we heated the empty microchamber pots and crucibles in the closed and clamped µ-CTE250 microchambers, at 200°C, for 10 minutes.

### Sample preparation

We made samples from 2 sources of synthetic hair, denoted as Source A and Source B. We purchased these popular and inexpensive brands from a local hair supply store, because they were recommended by our community/citizen-science research participants (Auguste et al, in preparation, 2020); because they were used in hair salons and also by lay consumers at home; and because one was flame retardant and the other was not flame retardant. Source A was a single package of synthetic hair made from the Kanekalon brand of polyester fiber; the packaging included a label that said “flame retardant” and recommendations for styling using hot water. Source B was a single package of synthetic hair; the packaging included language that said “Do not heat style while wearing. Avoid excess heat,” with no language about being flame retardant, so we classified it as a non-flame-retardant product. We prepared test samples from the 2 sources by cutting locks of hair from each source, approximately 0.3 g each. We weighed and logged each lock of hair and then placed each in a clean labeled porcelain crucible.

### Extraction procedures

**Figure 3.**
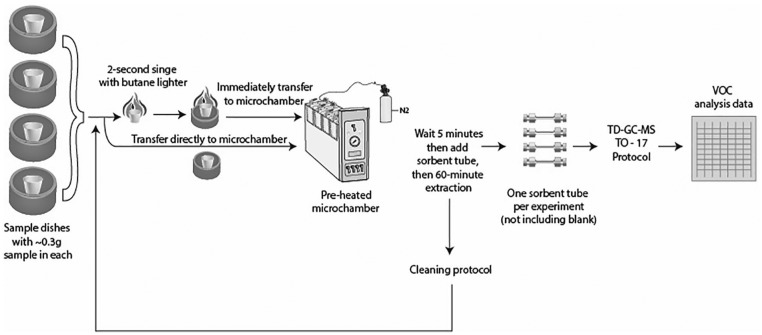
Sequence of steps in the method for collecting emissions, from singed and unsinged samples, into one sorbent tube per experiment, then analyzing through thermal desorption–gas chromatography–mass spectrometry (TD-GC-MS) protocol. A blank was collected before the first experiment.

Figure 3 shows the sequence of steps for our extraction procedures. Emissions were collected for 60 minutes for all samples, conservatively doubling the sampling time used in a combustion application case study documented by Markes International^22^ as an example. Before the first experiment, we collected emissions from a microchamber that held only an empty clean microchamber pot, into a sorbent tube that we logged as a blank.

For a non-singed run of samples from Source A and Source B, heated using the built-in heater, we pre-heated the microchambers to the target temperature. Then we placed the crucible containing the sample in a microchamber pot in a microchamber, closed and clamped the lid, allowed the sample to equilibrate for 5 minutes, and then attached the labeled Carbotrap 300 sorbent tube to the microchamber lid for 60 minutes of emission collection. We then removed the sorbent tube, capped it with a brass storage cap, logged it, and prepared it for shipment.

For a singed run of samples from Source A and Source B, also heated using the built-in heater, we pre-heated the microchambers to the target temperature. We used the butane lighter to singe the sample in a crucible for 2 seconds. We immediately transferred the crucible with smoldering sample into a microchamber pot in a microchamber, closed and clamped the lid, allowed the sample to sit for 5 minutes, and then attached the labeled Carbotrap 300 sorbent tube to the microchamber lid for 60 minutes of emission collection. We then removed the sorbent tube, capped it with a brass storage cap, logged it, and prepared it for shipment. Because we used N_2_ as a carrier gas, instead of oxygen, our extraction phase benefited from the residual smoldering of the singed samples, without triggering new combustion.

### Analysis

After all of the samples were run in the various scenarios, all of the labeled and logged sorbent tubes were prepared for shipment to an independent third-party laboratory that specializes in environmental analysis. That lab used EPA Method TO-17 to analyze the emission samples with TD-GC-MS.^23^ For this analysis method, compounds captured in the Carbotrap tube were released from the heated sorbent into the gas chromatograph and mass spectrometry system for separation, identification, and quantification of each compound. The specific system used was comprised of a Markes Analytical Thermal Desorption unit, an Agilent 5975 Mass Selective Detector, and an Agilent 7890A gas chromatograph with MS18 mass spectrometry unit.

## Results

An independent third-party laboratory provided results from the EPA Method TO-17 analysis. The case narrative noted that some of the compounds exceeded calibration range (discussed in section “Future Work”). In this study, we present the data from 4 complete experiment runs. Results from Source A samples, which were taken from flame-retardant synthetic hair, are shown in Tables 1 and 2. Results from Source B samples, which were taken from non-flame-retardant synthetic hair, are shown in Tables 3 and 4. The results from the laboratory are expressed as mass sampled per collection tube after 60 minutes of sampling had been completed.

**Table 1.** VOC emissions in sample tube after 60-minute sampling from Source A—flame-retardant synthetic hair, singed and heated at 250°C, weight 0.288 g.

CAS#	Compound	Result ng/tube
75-71-8	Dichlorodifluoromethane (CFC 12)	<1.0
74-87-3	Chloromethane	6300
76-14-2	1,2-Dichloro-1,1,2,2-tetrafluoroethane (CFC 114)	<1.0
75-01-4	Vinyl chloride	760
106-99-0	1,3-Butadiene	1500
75-00-3	Chloroethane	5400
64-17-5	Ethanol	<5.3
75-05-8	Acetonitrile	83 000
67-64-1	Acetone	250 000
75-69-4	Trichlorofluoromethane	<1.1
67-63-0	2-Propanol (isopropyl alcohol)	3100
75-35-4	1,1-Dichloroethene	100
75-09-2	Methylene chloride	190
76-13-1	Trichlorotrifluoroethane	<1.1
75-15-0	Carbon disulfide	62
156-60-5	trans-1,2-dichloroethene	<1.1
75-34-3	1,1-Dichloroethane	<1.0
1634-04-4	Methyl tert-butyl ether	<1.1
78-93-3	2-Butanone (MEK)	16 000
156-59-2	cis-1,2-Dichloroethene	<1.1
110-54-3	n-Hexane	<1.1
67-66-3	Chloroform	<1.1
109-99-9	Tetrahydrofuran (THF)	<1.1
107-06-2	1,2-Dichloroethane	3000
71-55-6	1,1,1-Trichloroethane	<1.1
71-43-2	Benzene	8700
56-23-5	Carbon tetrachloride	<1.1
110-82-7	Cyclohexane	<2.1
78-87-5	1,2-Dichloropropane	<1.1
75-27-4	Bromodichloromethane	<1.1
79-01-6	Trichloroethene	<1.1
123-91-1	1,4-Dioxane	<1.1
540-84-1	2,2,4-Trimethylpentane (isooctane)	<1.1
142-82-5	n-Heptane	<1.1
10061-01-5	cis-1,3-Dichloropropene	960
108-10-1	4-Methyl-2-pentanone	<2.1
10061-02-6	trans-1,3-Dichloropropene	880
79-00-5	1,1,2-Trichloroethane	<1.1
108-88-3	Toluene	1600
591-78-6	2-Hexanone	<1.1
124-48-1	Dibromochloromethane	<1.1
106-93-4	1,2-Dibromoethane	<1.1
111-65-9	n-Octane	<1.1
127-18-4	Tetrachloroethene	17
108-90-7	Chlorobenzene	76
100-41-4	Ethylbenzene	35
179601-23-1	m,p-Xylenes	130
75-25-2	Bromoform	<1.1
100-42-5	Styrene	120
95-47-6	o-Xylene	27
79-34-5	1,1,2,2-Tetrachloroethane	<1.1
98-82-8	Cumene	<1.1
108-67-8	1,3,5-Trimethylbenzene	<1.0
95-63-6	1,2,4-Trimethylbenzene	<1.1
541-73-1	1,3-Dichlorobenzene	15
106-46-7	1,4-Dichlorobenzene	15
95-50-1	1,2-Dichlorobenzene	<1.1
96-12-8	1,2-Dibromo-3-chloropropane	<1.1
120-82-1	1,2,4-Trichlorobenzene	<1.1
91-20-3	Naphthalene	710
87-68-3	Hexachlorobutadiene	<1.1

The CAS is the Chemical Abstract Service registry number.

**Table 2.** VOC emissions in sample tube after 60-minute sampling from Source A—flame-retardant synthetic hair, heated at 250°C but not singed, weight 0.307 g.

CAS#	Compound	Result ng/tube
75-71-8	Dichlorodifluoromethane (CFC 12)	<1.0
74-87-3	Chloromethane	20 000
76-14-2	1,2-Dichloro-1,1,2,2-tetrafluoroethane (CFC 114)	<1.0
75-01-4	Vinyl chloride	640
106-99-0	1,3-Butadiene	3400
75-00-3	Chloroethane	8800
64-17-5	Ethanol	<5.3
75-05-8	Acetonitrile	190 000
67-64-1	Acetone	340 000
75-69-4	Trichlorofluoromethane	<1.1
67-63-0	2-Propanol (isopropyl alcohol)	<2.1
75-35-4	1,1-Dichloroethene	320
75-09-2	Methylene chloride	490
76-13-1	Trichlorotrifluoroethane	<1.1
75-15-0	Carbon disulfide	240
156-60-5	trans-1,2-Dichloroethene	<1.1
75-34-3	1,1-Dichloroethane	22
1634-04-4	Methyl tert-butyl ether	<1.1
78-93-3	2-Butanone (MEK)	12 000
156-59-2	cis-1,2-Dichloroethene	<1.1
110-54-3	n-Hexane	74
67-66-3	Chloroform	<1.1
109-99-9	Tetrahydrofuran (THF)	<1.1
107-06-2	1,2-Dichloroethane	7600
71-55-6	1,1,1-Trichloroethane	<1.1
71-43-2	Benzene	8400
56-23-5	Carbon tetrachloride	<1.1
110-82-7	Cyclohexane	<2.1
78-87-5	1,2-Dichloropropane	<1.1
75-27-4	Bromodichloromethane	<1.1
79-01-6	Trichloroethene	<1.1
123-91-1	1,4-Dioxane	<1.1
540-84-1	2,2,4-Trimethylpentane (isooctane)	55
142-82-5	n-Heptane	<1.1
10061-01-5	cis-1,3-Dichloropropene	80
108-10-1	4-Methyl-2-pentanone	<2.1
10061-02-6	trans-1,3-Dichloropropene	120
79-00-5	1,1,2-Trichloroethane	<1.1
108-88-3	Toluene	350
591-78-6	2-Hexanone	<1.1
124-48-1	Dibromochloromethane	<1.1
106-93-4	1,2-Dibromoethane	<1.1
111-65-9	n-Octane	<1.1
127-18-4	Tetrachloroethene	8.4
108-90-7	Chlorobenzene	52
100-41-4	Ethylbenzene	9.7
179601-23-1	m,p-Xylenes	74
75-25-2	Bromoform	<1.1
100-42-5	Styrene	94
95-47-6	o-Xylene	16
79-34-5	1,1,2,2-Tetrachloroethane	<1.1
98-82-8	Cumene	<1.1
108-67-8	1,3,5-Trimethylbenzene	<1.0
95-63-6	1,2,4-Trimethylbenzene	<1.1
541-73-1	1,3-Dichlorobenzene	24
106-46-7	1,4-Dichlorobenzene	24
95-50-1	1,2-Dichlorobenzene	<1.1
96-12-8	1,2-Dibromo-3-chloropropane	<1.1
120-82-1	1,2,4-Trichlorobenzene	<1.1
91-20-3	Naphthalene	780
87-68-3	Hexachlorobutadiene	<1.1

The CAS is the Chemical Abstract Service registry number.

**Table 3.** VOC emissions in sample tube after 60-minute sampling from Source B—non-flame-retardant synthetic hair, singed and heated at 250°C, weight 0.316 g.

CAS#	Compound	Result ng/tube
75-71-8	Dichlorodifluoromethane (CFC 12)	<1.0
74-87-3	Chloromethane	960
76-14-2	1,2-Dichloro-1,1,2,2-tetrafluoroethane (CFC 114)	<1.0
75-01-4	Vinyl Chloride	<1.0
106-99-0	1,3-Butadiene	<1.1
75-00-3	Chloroethane	490
64-17-5	Ethanol	160
75-05-8	Acetonitrile	410
67-64-1	Acetone	6800
75-69-4	Trichlorofluoromethane	4.1
67-63-0	2-Propanol (isopropyl alcohol)	77
75-35-4	1,1-Dichloroethene	<1.1
75-09-2	Methylene chloride	29
76-13-1	Trichlorotrifluoroethane	<1.1
75-15-0	Carbon disulfide	11
156-60-5	trans-1,2-Dichloroethene	<1.1
75-34-3	1,1-Dichloroethane	<1.0
1634-04-4	Methyl tert-butyl ether	<1.1
78-93-3	2-Butanone (MEK)	100
156-59-2	cis-1,2-Dichloroethene	<1.1
110-54-3	n-Hexane	42
67-66-3	Chloroform	<1.1
109-99-9	Tetrahydrofuran (THF)	<1.1
107-06-2	1,2-Dichloroethane	21
71-55-6	1,1,1-Trichloroethane	<1.1
71-43-2	Benzene	490
56-23-5	Carbon tetrachloride	<1.1
110-82-7	Cyclohexane	<2.1
78-87-5	1,2-Dichloropropane	<1.1
75-27-4	Bromodichloromethane	<1.1
79-01-6	Trichloroethene	<1.1
123-91-1	1,4-Dioxane	320
540-84-1	2,2,4-Trimethylpentane (isooctane)	5.8
142-82-5	n-Heptane	17
10061-01-5	cis-1,3-Dichloropropene	<1.1
108-10-1	4-Methyl-2-pentanone	280
10061-02-6	trans-1,3-Dichloropropene	<1.1
79-00-5	1,1,2-Trichloroethane	<1.1
108-88-3	Toluene	150
591-78-6	2-Hexanone	<1.1
124-48-1	Dibromochloromethane	<1.1
106-93-4	1,2-Dibromoethane	<1.1
111-65-9	n-Octane	<1.1
127-18-4	Tetrachloroethene	<1.1
108-90-7	Chlorobenzene	<1.1
100-41-4	Ethylbenzene	17
179601-23-1	m,p-Xylenes	190
75-25-2	Bromoform	<1.1
100-42-5	Styrene	37
95-47-6	o-Xylene	28
79-34-5	1,1,2,2-Tetrachloroethane	<1.1
98-82-8	Cumene	<1.1
108-67-8	1,3,5-Trimethylbenzene	110
95-63-6	1,2,4-Trimethylbenzene	15
541-73-1	1,3-Dichlorobenzene	<1.1
106-46-7	1,4-Dichlorobenzene	10
95-50-1	1,2-Dichlorobenzene	5.5
96-12-8	1,2-Dibromo-3-chloropropane	<1.1
120-82-1	1,2,4-Trichlorobenzene	60
91-20-3	Naphthalene	94
87-68-3	Hexachlorobutadiene	<1.1

The CAS is the Chemical Abstract Service registry number.

**Table 4.** VOC emissions in sample tube after 60-minute sampling from Source B—non-flame-retardant synthetic hair, heated at 250°C but not singed, weight 0.297 g.

CAS#	Compound	Result ng/tube
75-71-8	Dichlorodifluoromethane (CFC 12)	11
74-87-3	Chloromethane	830
76-14-2	1,2-Dichloro-1,1,2,2-tetrafluoroethane (CFC 114)	1.4
75-01-4	Vinyl chloride	7.5
106-99-0	1,3-Butadiene	<1.1
75-00-3	Chloroethane	420
64-17-5	Ethanol	480
75-05-8	Acetonitrile	360
67-64-1	Acetone	710
75-69-4	Trichlorofluoromethane	1300
67-63-0	2-Propanol (isopropyl alcohol)	970
75-35-4	1,1-Dichloroethene	<1.1
75-09-2	Methylene chloride	440
76-13-1	Trichlorotrifluoroethane	2.8
75-15-0	Carbon disulfide	7.4
156-60-5	trans-1,2-Dichloroethene	<1.1
75-34-3	1,1-Dichloroethane	<1.0
1634-04-4	Methyl tert-butyl ether	<1.1
78-93-3	2-Butanone (MEK)	<1.1
156-59-2	cis-1,2-Dichloroethene	<1.1
110-54-3	n-Hexane	33
67-66-3	Chloroform	2.8
109-99-9	Tetrahydrofuran (THF)	8.6
107-06-2	1,2-Dichloroethane	20
71-55-6	1,1,1-Trichloroethane	<1.1
71-43-2	Benzene	280
56-23-5	Carbon tetrachloride	<1.1
110-82-7	Cyclohexane	<2.1
78-87-5	1,2-Dichloropropane	<1.1
75-27-4	Bromodichloromethane	<1.1
79-01-6	Trichloroethene	18
123-91-1	1,4-Dioxane	180
540-84-1	2,2,4-Trimethylpentane (isooctane)	<1.1
142-82-5	n-Heptane	24
10061-01-5	cis-1,3-Dichloropropene	<1.1
108-10-1	4-Methyl-2-pentanone	15
10061-02-6	trans-1,3-Dichloropropene	<1.1
79-00-5	1,1,2-Trichloroethane	<1.1
108-88-3	Toluene	290
591-78-6	2-Hexanone	<1.1
124-48-1	Dibromochloromethane	<1.1
106-93-4	1,2-Dibromoethane	<1.1
111-65-9	n-Octane	32
127-18-4	Tetrachloroethene	12
108-90-7	Chlorobenzene	9.6
100-41-4	Ethylbenzene	18
179601-23-1	m,p-Xylenes	140
75-25-2	Bromoform	<1.1
100-42-5	Styrene	23
95-47-6	o-Xylene	27
79-34-5	1,1,2,2-Tetrachloroethane	<1.1
98-82-8	Cumene	2.2
108-67-8	1,3,5-Trimethylbenzene	9.9
95-63-6	1,2,4-Trimethylbenzene	16
541-73-1	1,3-Dichlorobenzene	<1.1
106-46-7	1,4-Dichlorobenzene	21
95-50-1	1,2-Dichlorobenzene	14
96-12-8	1,2-Dibromo-3-chloropropane	<1.1
120-82-1	1,2,4-Trichlorobenzene	80
91-20-3	Naphthalene	14
87-68-3	Hexachlorobutadiene	<1.1

The CAS is the Chemical Abstract Service registry number.

### VOCs emitted from Source A, flame-retardant synthetic hair

Source A was sampled from a flame-retardant hair accessory product that was made from the Kanekalon synthetic fiber brand. Similar hair accessory products were made from the Toyokalon^®^ synthetic fiber brand. Both were described on their brand websites as “flame retardant.”^24,25^ Kanekalon was described as a flame-retardant polyester fiber made from a flame-retardant polyethylene-terephthalate (PET)—“an inherently flame retardant [*sic*] product in which the fiber resin itself contains the flame retardant [*sic*] ingredient.” Toyokalon was described as a polyvinyl chloride (PVC) fiber. We did not see any recommendations on either website encouraging users to singe the hair accessory products made from these fibers.

When Source A synthetic hair was burned, VOCs were detected, and the VOC species that we measured in the emissions are listed in Table 1. For example, acetone, acetonitrile, 2-butanone, and benzene were present in the emissions from singed Source A. When Source A synthetic hair was heated, without singeing, the VOC species that we measured in the emissions are listed in Table 2. Acetone, acetonitrile, chloromethane, 2-butanone, chloroethane, benzene, and 1,2-dichloroethane were all present in the emissions from heated Source A.

### VOCs emitted from Source B, non-flame-retardant synthetic hair

Source B was sampled from a hair accessory product that was not labeled with regard to flammability; the packaging included cautionary language, “Do not heat style while wearing. Avoid excess heat.” This hair accessory product was not labeled with details about its composition. Non-flame-retardant synthetic hair is typically made of non-flame-retardant PET fibers or from nylon synthetic fibers.^26^

When Source B synthetic hair was burned or heated, VOCs were detected, and the VOC species that we measured in the emissions are listed in Table 3. For example, acetone and chloromethane were present in the emissions from singed Source B. When Source B synthetic hair was heated, without singeing, the VOC species that we measured in the emissions are listed in Table 4. Trichlorofluoromethane, 2-propanol, chloromethane, and acetone were all present in the emissions from heated Source B.

Certain contextual information is important for using data from the TD-GC-MS analysis. Table 5 shows the list of 61 compounds on the EPA Method TO-17 Standard Compound List. These are the VOCs that the GC-MS is configured to recognize. Table 6 shows VOC emissions from our method blank.

**Table 5.** Standard compound list for EPA Method TO-17.

	CAS#	Compound	MRL ng/tube
1	75-71-8	Dichlorodifluoromethane (CFC 12)	0.5
2	74-87-3	Chloromethane	0.5
3	76-14-2	1,2-Dichloro-1,1,2,2-tetrafluoroethane (CFC 114)	0.5
4	75-01-4	Vinyl chloride	0.5
5	106-99-0	1,3-Butadiene	1.2
6	75-00-3	Chloroethane	1.0
7	64-17-5	Ethanol	5.1
8	75-05-8	Acetonitrile	2.0
9	67-64-1	Acetone	5.3
10	75-69-4	Trichlorofluoromethane	0.5
11	67-63-0	2-Propanol (isopropyl alcohol)	2.1
12	75-35-4	1,1-Dichloroethene	0.5
13	75-09-2	Methylene Chloride	0.5
14	76-13-1	Trichlorotrifluoroethane	0.5
15	75-15-0	Carbon disulfide	4.9
16	156-60-5	trans-1,2-Dichloroethene	0.5
17	75-34-3	1,1-Dichloroethane	0.5
18	1634-04-4	Methyl tert-butyl ether	0.5
19	78-93-3	2-Butanone (MEK)	1.1
20	156-59-2	cis-1,2-Dichloroethene	0.5
21	110-54-3	n-Hexane	0.5
22	67-66-3	Chloroform	0.5
23	109-99-9	Tetrahydrofuran (THF)	1.1
24	107-06-2	1,2-Dichloroethane	0.5
25	71-55-6	1,1,1-Trichloroethane	0.5
26	71-43-2	Benzene	2.2
27	56-23-5	Carbon tetrachloride	0.5
28	110-82-7	Cyclohexane	1.0
29	78-87-5	1,2-Dichloropropane	0.5
30	75-27-4	Bromodichloromethane	0.5
31	79-01-6	Trichloroethene	0.5
32	123-91-1	1,4-Dioxane	1.1
33	540-84-1	2,2,4-Trimethylpentane (isooctane)	0.5
34	142-82-5	n-Heptane	0.5
35	10061-01-5	cis-1,3-Dichloropropene	0.5
36	108-10-1	4-Methyl-2-pentanone	2.1
37	10061-02-6	trans-1,3-Dichloropropene	0.5
38	79-00-5	1,1,2-Trichloroethane	0.5
39	108-88-3	Toluene	0.5
40	591-78-6	2-Hexanone	1.1
41	124-48-1	Dibromochloromethane	0.5
42	106-93-4	1,2-Dibromoethane	0.5
43	111-65-9	n-Octane	0.5
44	127-18-4	Tetrachloroethene	0.5
45	108-90-7	Chlorobenzene	0.5
46	100-41-4	Ethylbenzene	0.5
47	179601-23-1	m,p-Xylenes	1.1
48	75-25-2	Bromoform	0.5
49	100-42-5	Styrene	0.6
50	95-47-6	o-Xylene	0.5
51	79-34-5	1,1,2,2-Tetrachloroethane	0.5
52	98-82-8	Cumene	0.5
53	108-67-8	1,3,5-Trimethylbenzene	0.5
54	95-63-6	1,2,4-Trimethylbenzene	0.5
55	541-73-1	1,3-Dichlorobenzene	0.6
56	106-46-7	1,4-Dichlorobenzene	0.5
57	95-50-1	1,2-Dichlorobenzene	0.5
58	96-12-8	1,2-Dibromo-3-chloropropane	1.1
59	120-82-1	1,2,4-Trichlorobenzene	0.6
60	91-20-3	Naphthalene	0.5
61	87-68-3	Hexachlorobutadiene	0.6

The CAS is the Chemical Abstract Service registry number. The MRL is the method reporting limit, lower limit for quantifying a target analyte, as provided by the EPA.^22^

**Table 6.** VOC emissions in method blank tube after 60-minute sampling of empty, clean microchamber.

CAS#	Compound	Result ng/tube
75-71-8	Dichlorodifluoromethane (CFC 12)	<1.0
74-87-3	Chloromethane	<1.0
76-14-2	1,2-Dichloro-1,1,2,2-tetrafluoroethane (CFC 114)	<1.0
75-01-4	Vinyl chloride	<1.0
106-99-0	1,3-Butadiene	<1.1
75-00-3	Chloroethane	<1.0
64-17-5	Ethanol	<5.3
75-05-8	Acetonitrile	<2.1
67-64-1	Acetone	<5.3
75-69-4	Trichlorofluoromethane	<1.1
67-63-0	2-Propanol (isopropyl alcohol)	<2.1
75-35-4	1,1-Dichloroethene	<1.1
75-09-2	Methylene chloride	<1.1
76-13-1	Trichlorotrifluoroethane	<1.1
75-15-0	Carbon disulfide	<5.3
156-60-5	trans-1,2-Dichloroethene	<1.1
75-34-3	1,1-Dichloroethane	<1.0
1634-04-4	Methyl tert-butyl ether	<1.1
78-93-3	2-Butanone (MEK)	<1.1
156-59-2	cis-1,2-Dichloroethene	<1.1
110-54-3	n-Hexane	<1.1
67-66-3	Chloroform	<1.1
109-99-9	Tetrahydrofuran (THF)	<1.1
107-06-2	1,2-Dichloroethane	<1.1
71-55-6	1,1,1-Trichloroethane	<1.1
71-43-2	Benzene	<2.1
56-23-5	Carbon tetrachloride	<1.1
110-82-7	Cyclohexane	<2.1
78-87-5	1,2-Dichloropropane	<1.1
75-27-4	Bromodichloromethane	<1.1
79-01-6	Trichloroethene	<1.1
123-91-1	1,4-Dioxane	<1.1
540-84-1	2,2,4-Trimethylpentane (isooctane)	<1.1
142-82-5	n-Heptane	<1.1
10061-01-5	cis-1,3-Dichloropropene	<1.1
108-10-1	4-Methyl-2-pentanone	<2.1
10061-02-6	trans-1,3-Dichloropropene	<1.1
79-00-5	1,1,2-Trichloroethane	<1.1
108-88-3	Toluene	<1.1
591-78-6	2-Hexanone	<1.1
124-48-1	Dibromochloromethane	<1.1
106-93-4	1,2-Dibromoethane	<1.1
111-65-9	n-Octane	<1.1
127-18-4	Tetrachloroethene	<1.1
108-90-7	Chlorobenzene	<1.1
100-41-4	Ethylbenzene	<1.1
179601-23-1	m,p-Xylenes	<2.1
75-25-2	Bromoform	<1.1
100-42-5	Styrene	<1.1
95-47-6	o-Xylene	<1.1

Abbreviation: CAS: Chemical Abstract Service.

## Discussion

Synthetic hair is used as a hair-styling accessory. It is typically manufactured from one or more of these materials: vinyl chloride, acrylonitrile, modacrylic, PET, PVC, polypropylene, and polyamide (nylon). Synthetic hair is used for hair styling across genders, ethnicities, and age groups to add length; add specific colors; add specific texture or curl; and to replace hair loss from baldness, chemotherapy, or alopecia. Hair styling that incorporates synthetic hair is an activity that is done informally in homes and also professionally in hair salons.

Figure 1 shows the hair-braiding and braid-burning sequence that stylists sometimes use. When the synthetic hair is singed, note the proximity to all participants’ breathing zones. The packaging of some synthetic hair accessory products explicitly states that the products are designed for low-heat styling (<177°C) or non-flame styling; some recommend dipping the hair into hot water or using rubber bands for styling. Nonetheless, research by Auguste et al (in preparation, 2020) has documented braid-burning/singeing as a popular practice in African American communities despite the product instructions. They documented that stylists sometimes chose to use flame-retardant synthetic hair to avoid generating excessive smoke during singeing (though there is some smoke from the butane lighter and a relatively small amount from the flame-retardant synthetic hair) and because it is perceived to be a safer, controlled burn than when singeing non-flame-retardant synthetic hair. In addition to or instead of singeing the synthetic hair at the tips of braids, users in a variety of communities sometimes choose to seal the tips or straighten/curl the hair using electric styling tools such as flatirons or curling irons (with temperatures up to 260°C) despite the product instructions.

Based on the data presented above, we have confirmed that VOCs are emitted when synthetic hair is singed, many of which are toxic and/or carcinogenic. Furthermore, we confirmed that VOCs are emitted when synthetic hair is heated at temperatures of 250°C, even if not singed. Our experimental design—with heat and singeing, and with heat and no singeing—gave us insights into VOC emissions from synthetic hair heated in 2 different ways that simulate aspects of the 2 use cases that are popular in the communities of concern. When designing the experiments, we kept in mind the 2 ways that are typically used, sustained heating and short singes, within the limitations of the instruments. The TD-GC-MS analysis provided us with the exact species of VOCs that are emitted in each tested scenario and their mass in ng per sample tube for those that have mass above the method reporting limit (MRL).

Health effects of some of the high-mass VOCs that we found in the heated synthetic hair emissions are summarized in Table 7, as provided by the US Agency for Toxic Substances & Disease Registry.^26^ Further work will be needed to model the exposure levels in typical usage scenarios.

**Table 7.** Health effects of 4 high-mass VOCs found in this study.

Compound	Health effects
Acetone	Moderate to high levels: headaches, skin and eye irritation, respiratory tract irritation From animal studies: birth defects, hormone disruption in males
Chloromethane	Very high levels: nervous system effects Lower levels: fatigue, confusion, vision effects Possible human carcinogen Animal studies: hormone effects
2-Butanone (MEK)	Inhalation causes irritation of skin, eyes, throat; birth defects; nervous system effects
Benzene	High levels: headaches, nervous system effects Long-term exposure causes damage to bone marrow and red blood cells, immune system, leukemia Known human carcinogen

Our study had certain limitations. We only tested 2 source products, although there are hundreds available on the market in the United States. The 2 products that we tested were recommended by consumers familiar with the braid-burning usage scenario that prompted this study, and the 2 products represented the flame-retardant and non-flame-retardant categories of the synthetic hair products. For this initial study, which aims to determine whether and which VOCs are emitted from heated synthetic hair, this positive finding from 2 popular and representative products answers the research questions and establishes a basis for further study.

Another limitation of our study is that we present the data from only 4 complete experiment runs—from emission collection into 4 sorbent tubes (a blank was also collected, before the first experiment) through emission analysis of those 5 tubes. Ideally, we would present multiple redundant experiment runs with datasets from each run. Our resources were too limited to conduct multiple redundant experiment runs. But to achieve the aims of this initial study and answer the research questions, we were able to confirm VOC emission from both types of synthetic hair. This positive finding establishes a basis for further study when we or others have resources available.

## Future Work

Based on our literature review and research, we concluded that this is an understudied area of indoor air pollutant emissions. Our positive findings in this study establish a basis for future study. Noting that some of the analysis results were flagged as having concentrations of compounds that exceeded calibration ranges, and noting the limitations presented above in the “Discussion” section, future work should include multiple runs of the same protocol with synthetic hair sourced from a wide variety of commercial products.

We recommend that future work includes additional detailed experiments in controlled test chambers or actual homes and salons. Also, modeling studies that accurately reflect all of the key components of the indoor usage scenarios for heated synthetic hair would be useful. Those key components should include modeling of the rooms where these usage scenarios may occur, such as homes and hair salons; modeling combustion times and proximity to human breathing zones; and modeling non-occupational reference exposure levels for children and adults. We also recommend that researchers create models to help measure exposure impact and VOC emissions of synthetic hair when worn in various hair styles, including hair styles that involve heating, singeing, combing, brushing, dipping into hot water, washing, blow drying, wearing for days, wearing for weeks, and wearing for months.

## Conclusions

The research questions that we sought to answer with this research were as follows: Are VOCs emitted from singed or heated synthetic hair? If so, what are the VOC species and relative masses identified in singed or heated synthetic hair? Our findings confirmed that VOCs are emitted from singed or heated synthetic hair. The specific species and emission concentrations differed for flame-retardant and non-flame-retardant synthetic hair. For flame-retardant synthetic hair, we found that the VOC emissions included acetone, acetonitrile, 2-butanone, benzene, chloromethane, chloroethane, and 1,2-dichloroethane. For non-flame-retardant synthetic hair, we found that the VOC emissions included acetone, acetonitrile, chloromethane, trichlorofluoromethane, and 2-propanol.

In verifying these VOC emissions in a laboratory setting, we confirmed the original observation of the community/citizen science participants from the indoor air quality research study. This work confirmed their informal findings with formal findings and identified the specific VOC species and their masses for future study.

## References

[1] Auguste D. A Data Science Approach to STEM (Science, Technology, Engineering and Math) Identity Research for African American Communities [dissertation]. Boulder: University of Colorado; 2019.

[2] Centers for Disease Control and Prevention (CDC). FastStats: Asthma. Atlanta, GA: CDC; 2017 https://www.cdc.gov/nchs/fastats/asthma.htm. Accessed March 1, 2019.

[3] Environmental Protection Agency (EPA). Protecting Children’s Environmental Health. Washington, DC: EPA; 2014 https://www.epa.gov/children. Accessed March 1, 2019.

[4] Environmental Protection Agency (EPA). Report to Congress on Indoor Air Quality: Volume 1: Federal Programs Addressing Indoor Air. Washington, DC: EPA; 1989 http://tinyurl.com/lsnr8os. Accessed March 1, 2019.

[5] Environmental Protection Agency (EPA). Report to Congress on Indoor Air Quality: Volume 2: Assessment and Control of Indoor Air Pollution. Washington, DC: EPA; 1989 http://tinyurl.com/lkrg3sc. Accessed March 1, 2019.

[6] Dinh TV Kim SY Son YS, et al Emission characteristics of VOCs emitted from consumer and commercial products and their ozone formation potential. Environ Sci Pollut Res Int. 2015;22:9345-9355. 25601614 10.1007/s11356-015-4092-8

[7] Nematollahi N Doronila A Mornane PJ Duan A Kolev SD Steinemann A. Volatile chemical emissions from fragranced baby products. Air Qual Atmos Health. 2018;11:785-790. 30147808 10.1007/s11869-018-0593-1 PMC6097056

[8] De Gennaro G de Gennaro L Mazzone A Porcelli F Tutino M Indoor air quality in hair salons: screening of volatile organic compounds and indicators based on health risk assessment. Atmos Environ. 2014;83:119-126.

[9] Lamplugh A Harries M Xiang F Trinh J Hecobian A Montoya LD. Occupational exposure to volatile organic compounds and health risks in Colorado nail salons. Environ Pollut. 2019;249:518-526. doi:10.1016/j.envpol.2019.03.086. 30933751

[10] Zhong L Batterman S Milando CW. VOC sources and exposures in nail salons: a pilot study in Michigan, USA. Int Arch Occup Environ Health. 2019;92:141-153. doi:10.1007/s00420-018-1353-0. 30276513 PMC6325001

[11] Hadei M Hopke PK Shahsavani A, et al Indoor concentrations of VOCs in beauty salons: association with cosmetic practices and health risk assessment. J Occup Med Toxicol. 2018;13:30. doi:10.1186/s12995-018-0213-x. 30275872 PMC6161385

[12] Delfino RJ Gong H Linn WS Hu Y Pellizzari ED Respiratory symptoms and peak expiratory flow in children with asthma in relation to volatile organic compounds in exhaled breath and ambient air. J Expo Anal Environ Epidemiol. 2003;13:348-363. doi:10.1038/sj.jea.7500287. 12973363

[13] Boyle EB Viet SM Wright DJ, et al Assessment of exposure to VOCs among pregnant women in the National Children’s Study. Int J Environ Res Public Health. 2016;13:376. doi:10.3390/ijerph13040376. 27043585 PMC4847038

[14] Hoang T Castorina R Gaspar F, et al VOC exposures in California early childhood education environments. Indoor Air. 2017;27:609–621. doi:10.1111/ina.12340. 27659059

[15] Tanus A Oliveira CCC Villarreal DJV Sanchez FA Dias MF. Black women’s hair: the main scalp dermatoses and aesthetic practices in women of African ethnicity. An Bras Dermatol. 2014;90:450-465. 10.1590/abd1806-4841.20152845 PMC4560533 26375213

[16] Chin JY Godwin C Parker E, et al Levels and sources of volatile organic compounds in homes of children with asthma. Indoor Air. 2014;24:403-415. 24329990 10.1111/ina.12086 PMC4057989

[17] Martin MA Thomas AM Mosnaim G Greve M Swider SM Rothschild SK. Home asthma triggers: barriers to asthma control in Chicago Puerto Rican children. J Health Care Poor Underserved. 2013;24:813-827. 23728047 10.1353/hpu.2013.0073 PMC8358823

[18] Kamarulzaman NH Le-Minh N Stuetz RM. Identification of VOCs from natural rubber by different headspace techniques coupled using GC-MS. Talanta. 2019;191:535-544. 30262095 10.1016/j.talanta.2018.09.019

[19] Schieweck A Bock MC. Emissions from low-VOC and zero-VOC paints: valuable alternatives to conventional formulations also for use in sensitive environments. Build Environ. 2015;85:243-252.

[20] Yu CF Crump DR. Small chamber tests for measurement of VOC emissions from flooring adhesives. Indoor Built Environ. 2003;12:299-310.

[21] Markes International. M-CTE250(T)(i)™ Micro-Chamber/Thermal Extractor Operator Manual Version 1.14. Llantrisant: Markes International; 2014.

[22] Markes International. Application Note 100: Comprehensive Analysis of Aroma Compounds Released From Incense Sticks Using TD-GC-MS. Llantrisant: Markes International; 2012.

[23] Environmental Protection Agency (EPA). Compendium of Methods for the Determination of Toxic Organic Compounds in Ambient Air: Compendium Method TO-17 Determination of Volatile Organic Compounds in Ambient Air Using Active Sampling onto Sorbent Tubes. Cincinnati, OH: U.S. Environmental Protection Agency; 1999.

[24] Kanekalon-hair.com. About Kanekalon website. http://www.kanekalon-hair.com/en/about.html. Accessed January 15, 2019.

[25] Toyokalon.com. What is Toyokalon website. https://www.toyokalon.com/about-us. Accessed January 15, 2019.

[26] ATSDR.CDC.gov. ATSDR (Agency for toxic substances & disease registry) toxic substances portal website. https://www.atsdr.cdc.gov/substances/index.asp. Accessed September 9, 2019.

